# Abstracts From the First Annual JADPRO Live Transforming Oncology Practice Meeting

**Published:** 2014-11-01

##  

LOEWS ROYAL PACIFIC HOTEL AT UNIVERSAL, ORLANDO, FLORIDA OCTOBER 30 – NOVEMBER 2, 2014 

## JL202. PD-1 Pathway Immune Checkpoint Inhibitors: The Next Wave in Immunotherapy for Melanoma

Rajni Kannan, BS, MS, RN, ANP-BC, and Kathleen Madden, RN, MSN, FNP-BC, AOCNP®; NYU Cancer Institute, NYU Langone Medical Center

Novel immune checkpoint inhibitors are changing the treatment options for advanced melanoma. Advanced practitioners may be familiar with ipilimumab, which inhibits the cytotoxic T-lymphocyte-associated antigen 4 (CTLA-4) pathway. The next generation of immune checkpoint inhibitors is comprised of agents targeting the programmed death-1 (PD-1) pathway. Pembrolizumab, a PD-1 inhibitor, was recently approved for the treatment of patients who progressed on or after ipilimumab therapy. Nivolumab, also a PD-1 inhibitor, is approved in Japan for the treatment of unresectable melanoma and was granted priority review status by the FDA. Both CTLA-4 and PD-1 have distinct but complimentary roles in regulating immune responses. Whereas CTLA-4 prevents T cell proliferation at the start of an immune response, PD-1 is thought to contribute to T cell exhaustion in peripheral tissues, including tumors. By blocking these pathways, antitumor T cell responses can be restored, leading to elimination of the cancer cells by the patient’s own immune system. In a phase Ib registrational trial of pembrolizumab, the response rate was 26% and the 1-year survival rate was 58%–63% (dose-dependent). In the first randomized, controlled phase III trial of a PD-1 inhibitor, nivolumab, the response rate was 32% versus 11% with chemotherapy.2 In a phase 1 nivolumab trial, the response rate was 31% and 1-/2-year survival was 62%/43%. A phase I trial combining ipilimumab with nivolumab showed increased efficacy (response rate: 42%; 1-/2-year survival: 85%/79%) than seen previously with monotherapy, suggesting synergy with dual blockade of both pathways. Unlike chemotherapy and targeted agents which act directly on the tumor cells, restoring antitumor responses with immune checkpoint blockade can be associated with novel response patterns and select immunologic adverse events (AEs) which are related to the mechanism of action. Practitioners should be aware that first responses may occur several months after initiating treatment, and, in some patients, show initial evidence of tumor progression, termed "pseudoprogression." Practitioners must also be vigilant for AEs with immune etiologies which need prompt diagnosis and management. PD-1 pathway inhibitors appear to have similar types of select immunologic AEs as ipilimumab, but with lower incidences and typically of lower grade; the most common AEs are fatigue, and typically mild skin and gastrointestinal events. Select grade 3–4 AEs have also been reported, including endocrinopathies, hepatitis, and pneumonitis (each ≤ 2%). Proper education of the healthcare team and patient is key to optimal management of patients receiving immune checkpoint inhibitors.

## JL203. Clinical Practice: Hypercalcemia of Malignancy

Steve Malangone, MSN, NP-C; Department of Hematology-Oncology, The University of Arizona Cancer Center, North Campus

Hypercalcemia is a common paraneoplastic syndrome associated with a variety of malignancies including lymphomas, aerodigestive, uterine, endometrial, breast, neuroendocrine, cervical, and renal cell carcinomas. The incidence of hypercalcemia in the cancer population is noted to be as high as 20-30%. Hypercalcemia associated with malignancy is associated with significant morbidity, including progressive cognitive dysfunction, dehydration, and acute renal failure. Sequelae of uncorrected hypercalcemia include dysrhythmia, coma, and death, with reports of as many as 50% mortality within 30 days of diagnosis. The advanced practitioner in oncology must be adequately prepared to identify and intervene appropriately in order to improve patient outcomes. The poster will present the clinical case of a patient with hypercalcemia (serum corrected calcium 14.2 mg/dL) in the setting of progression of well differentiated metastatic neuroendocrine tumor who presented with dehydration, progressive muscle weakness, lethargy, and confusion. The poster will describe clinical presentation, including laboratory values revealing elevated parathyroid-related hormone (PTHrP), symptoms including lethargy, confusion, and dehydration. Ultimately, the patient responded to aggressive hydration and diuresis, administration of intravenous bisphosphonates, and effective systemic management of disease. More broadly, the poster will include an overview of the presentation, etiology, therapeutic options, and practical considerations in managing this important oncologic syndrome. Diagnostic evaluation and consideration of differential diagnoses with an emphasis of various etiologies (osteolytic, humoral, increased calcitriol, and PTH secreting malignancies), general supportive measures, hydration, pharmacologic management, monitoring, and goals of therapy are included. The underlying pathophysiology of hypercaclemia of malignancy includes humoral, osteolytic, increased calcitriol, and parathyroid hormone (PTH) secreting malignancies. The most common mechanism (80%) is humoral hypercalcemia (HHM), in which tumor cells secrete ectopic PTHrP. In this disorder, PTHrP binds PTH receptors, inhibiting the action of osteoclasts and stimulating osteoblasts in the bone, and promoting renal tubular calcium reabsorption. Osteolytic hypercalcemia is directly related to the excessive release of calcium by osteoclasts in patients with osteolytic lesions. Increased calcitrol is related to increased conversion of inactive vitamin D to 1,25-dihydroxyvitamin D (calcitriol). This conversion, which is typically tightly regulated in the kidney through PTH and serum calcium concentration, occurs in an uninhibited fashion by way of PTH independent extrarenal malignant lymphocytes in the setting of lymphoma. Finally and rarely, PTH may be secreted, leading to hypercalcemia through the actions of PTH. Patients with mild hypercalcemia (serum calcium 10.5-12 mg/dL) can present with constipation, fatigue, and depression. Those with moderate hypercalcemia (12 to 14 mg/d) can develop polydipsia, polyuria, clinical dehydration, muscle weakness, and changes in mental status. Those with severe hypercalcemia can develop severe form of these symptoms. Diagnostic evaluation includes measurement of serum calcium, ionized calcium, PTH, and PTHrP. Patients with hypercalcemia of malignancy, secretion of endogenous parathyroid hormone (PTH) itself is suppressed by the PTHrP-mediated hypercalcemia. Goals of management include reduction in serum calcium, supportive management, and correction of underlying cause. The mainstay of therapy is hydration and calciuresis with IV normal saline and furosemide and intravenous bisphosphonate therapy. Ultimately, hypercalcemia is a clinical indication of progressive disease. Hypercalcemia of malignancy typically responds to control of systemic disease, and thus therapy to manage disease if available, should be initiated as soon as possible.

## JL204. Efficacy of a Transdermal Granisetron Patch in Controlling Chemotherapy-Induced Nausea and Vomiting (CINV) in Head and Neck Cancer Patients

Deborah Braccia RN, MPA, Ph, Prostrakan, Inc., Bridgewater, NJ, and Gary Shelton DNP(c), MSN, NP, ANP-BC, AOCNP, NYU Langone’s Laura and Isaac Perlmutter Cancer, New York, NY

*Objective*: To examine the efficacy of a transdermal granisetron patch for controlling CINV in head and neck cancer patients. *Significance*: Head and neck cancer patients can experience mechanical obstruction or dysphagia making adherence to oral medications, including common oral antiemetics, very difficult. A granisetron transdermal system (GTS) has been shown to be as effective as oral granisetron in controlling CINV across multiple tumor types. This post-hoc analysis specifically examined the efficacy and safety of GTS in difficult to treat head and neck cancer patients. *Purpose*: To compare the rates of complete control (CC; no vomiting, mild nausea, no rescue medication), complete response (CR; no vomiting, no rescue medication), need for rescue medication, and patient-reported assessment in head and neck cancer patients using either GTS or oral granisetron. *Methods*: A randomized, phase 3 study has been published comparing GTS (7 day application) to oral granisetron (2 mg/day) in patients receiving either moderately or highly emetogenic chemotherapy for 3-5 days. Data for this analysis were limited to patients with head and neck primary tumors. *Results*: 71 patients (38 GTS, 33 oral granisetron) were included. The CC rate of 66% and CR rate of 68% in the GTS group were similar to rates in the overall population. There was no difference in CC, CR, and use of rescue medication between GTS and oral granisetron (*p* = .94, .91, and .57, respectively). Patient assessment of overall response to therapy was not different between arms (*p* = .26). GTS was well tolerated, and treatment-related adverse events were mild. *Discussion*: This retrospective analysis suggests GTS may be an appropriate option for prevention of CINV in head and neck cancer patients at high risk of dysphagia treated with chemotherapy. Most curriculums do not include integrative modalities.

## JL205. Differences in Metabolite Activity of the Selective Estrogen Receptor Modulators Tamoxifen and Toremifene: Clinical Implications for Patients with Hormone Receptor Positive Breast Cancer

Marcelle Kaplan, RN, MS, AOCN, CBCN, Adelphi University College of Nursing and Public Health, Garden City, NY, and Deborah Braccia, RN, MPA, PhD, Prostrakan Inc. Bridgewater, NJ

*Objective*: To review published data on the metabolism of toremifene and assess any implications for treatment decisions. Significance: The use of selective estrogen receptor modulators (SERMs) forms part of the treatment backbone of hormone receptor positive breast cancer. Studies have shown that tamoxifen and toremifene, the two SERMs approved in the treatment of hormone receptor positive breast cancer, have similar efficacy and safety profiles. Although tamoxifen metabolism has been extensively studied, there has been limited information regarding toremifene metabolism and any potential implications for practice. *Purpose*: To review recent data on the metabolism of toremifene as well as significant drug-drug interactions that may impact treatment decisions. *Methods*: A literature review was conducted using www.pubmed.com. Toremifene, metabolism, Fareston, cytochrome P450, and CYP2D6 were used as search terms. Results: Toremifene is thought to be active in its parent form, and is mainly metabolized in the liver by CYP3A4. In contrast, tamoxifen requires metabolism by CYP2D6 to be converted to its biologically active 4-hyroxyl metabolites. Data suggest that potent CYP2D6 inhibitors, such as certain selective serotonin reuptake inhibitors (SSRIs), can result in alterations in plasma concentrations of the 4-hydroxyl metabolites of tamoxifen. Although published studies on the use of SSRIs and outcome of adjuvant therapy with tamoxifen remain discordant, NCCN guidelines currently recommend against coadministration of strong inhibitors of CYP2D6 and tamoxifen. A series of recent studies directly compared the metabolic activity of toremifene and tamoxifen in human liver microsomes and showed that metabolism of toremifene was unaffected by the presence of potent CYP2D6 inhibitors or by CYP2D6 genetic status. However, metabolism of tamoxifen was significantly altered by CYP2D6 inhibitors and CYP2D6 genetic status.. Concentrations of the active form of toremifene were unaffected by SSRIs or potential variation in CYP2D6 genetic status. *Discussion*: Although variations in CYP2D6 metabolic activity cause differences in plasma concentrations of active tamoxifen metabolites, the debate regarding the clinical sequelae of the CYP2D6 alterations and outcomes in patients taking tamoxifen is ongoing. Potential drug-drug interactions with SSRIs may alter the efficacy of tamoxifen and thereby inform treatment choices. Toremifene is unaffected by coadministration with SSRIs and represents a well-studied SERM option for treating patients with estrogen-positive breast cancer where CYP2D6 inhibition may be of concern.

## JL206. ONc-PoWER: Oncology Nurse Practitioner Web Education Resource

Margaret Quinn Rosenzweig, PhD, FNP-BC, AOCNP, Sara Klein, MS, Rose Hoffmann, PhD, RN, University of Pittsburgh School of Nursing

*Background*: Nurse practitioners (NP) new to cancer care are entering practice without any standardized oncology curriculum. This gap in knowledge can lead to poor patient outcomes, risk management vulnerabilities, and high clinician attrition. Existing online continuing education programs use the traditional Power Point teaching method. The ONc-PoWER course follows an avatar NP, new to oncology, as she encounters common clinical dilemmas in cancer care. Content is presented and then quickly applied to "real" case based situations. The interactive course provides constant feedback and motivation to the learner. The purpose of this study is to describe the ONc-PoWER course curriculum. *Method*: The Oncology Nurse Practitioner Web Education Resource (ONc-PoWER) is an online course developed and funded as education specifically for NPs in their first year of oncology practice paired with an onsite mentor (physician, nurse practitioner or physician assistant). The course was developed based on the Oncology Nursing Society’s Competencies for Entry to Practice. The course consists of 5 interactive modules: (1) the new patient visit, (2) presenting a patient with cancer, (3) cancer visits across the continuum of care, (4) palliative and hospice care, and (5) self-care and professional development. The course also has an evaluation for the NP or mentor for applying the content of the course to clinical practice. The course is being offered free of charge nationally to 100 NPs for a 6-month period. The NP can earn continuing education credit for content completion. *Results*: The primary outcome is the NPs perceived level of knowledge and confidence in the delivery of cancer care pre and post the ONc-PoWER curriculum. Onsite mentor will provide evaluation. The NPs attention to patient reported outcomes will also be measured pre and post curriculum. Curriculum overall will be assessed by the NPs and onsite mentors. The course just opened to formal evaluation, preliminary data consists of senior NP’s "testers" of the program. *Conclusion*: The course is available through the University of Pittsburgh Blackboard Learning System with an assigned instructor to monitor, organize and track the student’s progress through the course.

## JL207. Decision Making Factors and Outcomes: Cancer Clinical Trials

Barbara A. Biedrzycki, PhD, CRNP, AOCNP, Sidney Kimmel Comprehensive Cancer Center at Johns Hopkins, Baltimore, MD

*Background*: The Research Decision Making Model guided this completed research on decision making for cancer clinical trial participation. The Research Decision Model was based on Bowling and Ebrahim’s Model for Treatment Decision Making and modified based on a systematic review of the literature. One hundred ninety seven patients with advanced gastrointestinal cancers, pancreas and colorectal were eligible and completed participation in this descriptive, cross-sectional designed research. Mailed surveys and medical record review methods were used to identify disease context and sociodemographic factors, patient preferences for research decision control, hope, quality of life, and trust that influence the decision to participate or not to participate in a cancer clinical trial, and the satisfaction with the decision made. The self-reported survey responses and medical records review provided the data for descriptive and multiple logistic regression analyses. The three variables that predicted cancer clinical trial participation were: a cancer stage of less than 4; more hope as measured by the Herth Hope Index; and more trust in the health care system as measured by the Health Care System Distrust Scale. Furthermore, the preferred decision making style for cancer clinical trial participation is shared or collaborative decision making. This is a previously undiscovered factor for cancer clinical trial participation decision making, and one that provides unique opportunities for oncology advanced practitioners.

## JL208. Medical Orders for Life Sustaining Treatment: Making the Most Out of the MOLSTs

Barbara A. Biedrzycki, PhD, CRNP, AOCNP, Sidney Kimmel Comprehensive Cancer Center at Johns Hopkins, Baltimore, MD

There are several names given across the United States for the orders that direct end of care life. In some states, it is called Medical Orders for Life Sustaining Treatment (MOLST), in other states, Provider (or Physician) Orders for Life Sustaining Treatment (POLST). No matter the name the foundation is the same. The creation of these orders promote guidance on preferred end of life care. Regulations vary among the states regarding the logistics of obtaining these orders. This presentation will provide data on the similarities and diversities among orders for life sustaining treatments, as well as highlight the essential role of advanced practitioners in oncology in optimizing the discussion experience for the patient, and the order’s value to the health care system.

## JL209. Vaccinations Post Hematopoietic Stem Cell Transplantation

Kathleen Leonard, RN, NP-C, AOCNP, NYU Langone Medical Center, New York, NY

*Purpose*: The purpose of this poster presentation is to educate advanced practice professionals regarding vaccinations post hematopoietic stem cell transplantation and to focus on the standardization and implementation of vaccinations post hematopoietic stem cell transplantation. *Background*: Hematopoietic stem cell transplantation (HSCT) is the process by which stem cells that are destroyed by high doses of chemotherapy and radiation are replaced by healthy stem cells that have been harvested from bone marrow, peripheral blood or umbilical cord blood. The two major types of transplants are: autologous; in which patients receive their own stem cells and allogeneic; in which the patient receives stem cells from that of another person who may or may not be related. Patients that undergo hematopoietic stem cell transplantation become severely immune-compromised post transplantation and are at risk of developing bacterial and viral infections. HSCT recipients lose protective immunity to vaccine preventable diseases and this becomes a significant cause of re-hospitalization, morbidity and mortality. Inactivated vaccines are safe for the HSCT population. These include diphtheria-pertussis vaccine, haemophilus influenza vaccine, inactivated polio vaccine, hepatitis B vaccine, and pneumococcal vaccine, given at recommended scheduled times. *Discussion and Implications for Advanced Practitioners*: The literature clearly dictates the importance of re-immunization in the post hematopoietic stem cell transplantation recipients. The global aim is to eliminate preventable infections, decrease re-hospitalizations, decrease morbidity and mortality and ultimately avoid a public health crisis. Specifically a standardization of education and documentation of vaccinations is beneficial in the theme of quality care and safety in this patient population. It is imperative that the advance practice professional working with this population have a solid knowledge base in understanding the needs of these patients. The importance of patient education regarding immunization must be clear and concise and reinforced during scheduled follow up visits. Implementation of a post stem cell transplant vaccination program is necessary for this patient population.

## JL210. Acupuncture for the Management of Hot Flashes in Breast Cancer Survivors

Hollis McClellan Misiewicz, DNP, CRNP, AOCN, Mercy Medical Center

In the United States more women are diagnosed with breast cancer than any other type of cancer. As survival for these women improve health care practitioners must address long-term problems secondary to cancer therapy. The development of severe hot flashes, a common sequelae of treatment, can significantly affect quality of life. Many popular hot flash treatments are ineffective or contraindicated for breast cancer survivors. Research supports the use of acupuncture as an effective, safe treatment for minimizing hot flashes in breast cancer survivors. *Purpose/Design*: The purpose of this evidence-based practice project was to determine if acupuncture is effective at decreasing the number and severity of hot flashes and improving sleep in breast cancer survivors. Twenty women were recruited for this study with a quasi-experimental pretest-posttest design utilizing the Pittsburgh Sleep Quality Index and hot flash diaries. No control group was used as the study was designed to determine an individual’s response to acupuncture therapy. Twenty participants were recruited for the study. *Results*: Paired t-test revealed significant improvement in night time hot flash frequency and severity, daytime hot flash severity, and sleep quality. Participants’ descriptions of acupuncture encompassed three themes; acupuncture as (1) effective, (2) relaxing, and (3) painful at times. *Benefits/Limitations*: The pretest-posttest design of the study allowed for evaluation of individual patients to determine the effectiveness of acupuncture for controlling hot flashes and improving sleep. Limitations of the study that threatened internal validity included the lack of a control group, self-selection of participants which could introduce bias, and the high attrition rate of 50%. Limitations that affected external validity included participants selected from a single site, small sample size, and the use of one acupuncturist. *Conclusion*: Acupuncture can be effective in reducing the severity and frequency of hot flashes in breast cancer survivors. Future research should address the use of acupuncture with cancer populations other than breast cancer survivors, such as women with gynecological cancers or men with prostate cancer. Research addressing the influence of socioeconomic status and educational level on the use of acupuncture could provide findings that would assist health care providers give patient-specific information and recommendations about acupuncture. Advanced oncology practitioners are in a unique position to address the long-term problems secondary to chemotherapy that cancer survivors face. Evidence-based recommendations for management of the sequelae of chemotherapy improve the quality of care provided to cancer survivors.

## JL211. PD-1 Immune Checkpoint Inhibitors: An Exciting New Approach for the Treatment of Non-Small Cell Lung Cancer

Colleen Lewis, MSN, ANP-BC, AOCNP, Emory Winship Cancer Institute

Programmed death-1 (PD-1) immune checkpoint inhibitors are novel immuno-oncology agents being developed for the treatment of advanced non-small cell lung cancer (NSCLC) and other cancers. Clinical trial data have shown promising antitumor activity in patients with NSCLC, and approval of PD-1 immune checkpoint inhibitors in lung cancer is expected soon. Immune checkpoint inhibitors that target the PD-1 and CTLA-4 pathways are furthest along in clinical development. Unlike chemotherapy or targeted agents which act directly on the tumor to stop proliferation or induce tumor cell death, immuno-oncology agents are designed to stimulate a patient’s own immune system to eliminate tumors. When PD-1 on a T cell binds to one of its ligands, PD-L1 or PD-L2, T-cell activation is inhibited, suppressing T-cell attack. Tumors can express PD-L1 as a mechanism to avoid antitumor T-cell immune responses. PD-1 immune checkpoint inhibitors are designed to prevent PD-1 pathway mediated suppression of T-cell activity and restore antitumor immune responses. Initial phase 1 data with immune checkpoint inhibitors against PD-1 or PD-L1 have shown response rates in patients with advanced or metastatic NSCLC of 10–23% (across agents), and 1- and 2-year survival rates of 42% and 24% (nivolumab). Responses with nivolumab were seen across a broad array of NSCLC patient populations, regardless of histology or mutational status, and including heavily pretreated and older patients. The incidence of grade ≥3 adverse events (AEs) in trials of PD-1 immune checkpoint inhibitors ranged from 10–14%. The most commonly-reported AE with PD-1 agents is fatigue. As a result of their mechanism of action of stimulating immune responses, immune checkpoint inhibitors may be associated with select AEs with immunologic etiologies. Practitioners must thoroughly assess baseline AEs and be vigilant for select treatment emergent immunologic AEs which require prompt diagnosis and management. These include skin and gastrointestinal AEs (typically mild), and endocrine, hepatic, renal, and respiratory AEs, including pneumonitis. Most select AEs in clinical trials of nivolumab were effectively managed with treatment interruption and/or corticosteroid or other immunosuppressive agents. Endocrinopathies may require permanent hormone replacement therapy. A specialist consult (gastroenterologist, pulmonologist, endocrinologist) may be required in some cases. Infusion-related AEs with immune checkpoint inhibitors were uncommon in initial trials with NSCLC patients (≤5% all grades; ≤1% grade 3–4). A good understanding of the clinical profile of PD-1 pathway inhibitors will be instrumental in helping advanced practitioners manage patients who will receive these treatments in the near future.

